# Multidisciplinary Community-Based Investigation of a COVID-19 Outbreak Among Marshallese and Hispanic/Latino Communities — Benton and Washington Counties, Arkansas, March–June 2020

**DOI:** 10.15585/mmwr.mm6948a2

**Published:** 2020-12-04

**Authors:** Katherine E. Center, Juliana Da Silva, Angela L. Hernandez, Kristyn Vang, Daniel W. Martin, Jerry Mazurek, Emily A. Lilo, Nichole K. Zimmerman, Elisabeth Krow-Lucal, Ellsworth M. Campbell, Janet V. Cowins, Chastity Walker, Kenneth L. Dominguez, Bill Gallo, Jayleen K.L. Gunn, Donald McCormick, Cassie Cochran, Michelle R. Smith, Jennifer A. Dillaha, Allison E. James

**Affiliations:** ^1^CDC COVID-19 Response Team; ^2^Arkansas Department of Health; ^3^Epidemic Intelligence Service, CDC.

By June 2020, Marshallese and Hispanic or Latino (Hispanic) persons in Benton and Washington counties of Arkansas had received a disproportionately high number of diagnoses of coronavirus disease 2019 (COVID-19). Despite representing approximately 19% of these counties’ populations (*1*), Marshallese and Hispanic persons accounted for 64% of COVID-19 cases and 57% of COVID-19–associated deaths. Analyses of surveillance data, focus group discussions, and key-informant interviews were conducted to identify challenges and propose strategies for interrupting transmission of SARS-CoV-2, the virus that causes COVID-19. Challenges included limited native-language health messaging, high household occupancy, high employment rate in the poultry processing industry, mistrust of the medical system, and changing COVID-19 guidance. Reducing the COVID-19 incidence among communities that suffer disproportionately from COVID-19 requires strengthening the coordination of public health, health care, and community stakeholders to provide culturally and linguistically tailored public health education, community-based prevention activities, case management, care navigation, and service linkage.

All laboratory-confirmed COVID-19 cases in Benton and Washington counties in the Arkansas Department of Health (ADH) database reported during March 11–June 13, 2020, were included in these analyses. Community engagement was conducted during June 15–July 3, 2020, to identify challenges to interrupting SARS-CoV-2 transmission. Based on information from the community and ADH, all Native Hawaiian/Pacific Islander persons in Benton and Washington counties were considered Marshallese. Marshallese persons come from the Republic of the Marshall Islands, a sovereign nation and part of the Compact of Free Association. The Marshallese population has higher rates of some adverse health outcomes because of long-standing systemic factors, including poverty, poor access to care, and a nuclear bomb testing program during the Cold War (*2*).

Three focus groups with Marshallese community members (26 total participants) and three with Hispanic community members (30 total participants) were conducted to understand drivers of transmission and determine community-level perspectives and needs related to COVID-19. Separate focus groups including students, community members, and faith leaders were held online and in-person in English, Spanish, and Marshallese. Two churches and 21 businesses were visited across both counties, and key-informant interviews were conducted with Marshallese and Hispanic community leaders. Notes were taken during focus group discussions and key-informant interviews; next, two CDC team members reached consensus of the themes presented by the Hispanic and Marshallese communities independently. Themes were reviewed and brought to consensus with other team members present at the activity (Box). Quantitative analyses were conducted using SAS (version 9.4; SAS Institute). This activity was reviewed by CDC and was conducted consistent with applicable federal law and CDC policy.*

BOX Themes* identified from six focus group discussions with Marshallese and Hispanic or Latino communities of Benton and Washington Counties, Arkansas, June 15–July 3, 2020^†^

**Concern about family and community**

- Confusion and anxiety about testing and getting results
- Actively involved in COVID-19 education, mitigation, and support services
- Assembled task forces and were tracking cases and deaths in their populations

**Need for increased understanding and awareness about all aspects of prevention, testing, isolation, and treatment of COVID-19**

- Inconsistent messages from authorities, reopening the state, and communication barriers led to miscommunications and misunderstandings
- Need more knowledge of health care systems, resources, and support services to access and navigate
- Need more translated communication and resources describing
  - Modes of transmission of COVID-19
  - How specific prevention behaviors decrease COVID-19 risk
  - Factors that increase risk for COVID-19–associated complications or death
  - Testing, including how to get results
  - When to seek emergency care
- Messaging needs to come from local sources in a variety of ways
- Messaging needs to be repeated

**Actual and perceived barriers to testing, health care, and support services**

- Lack of knowledge on availability of resources, both typical and COVID-19–specific
- Lack of knowledge or understanding on how to access resources that are available
- Language barriers
- Lack of primary health care (affects health as well as knowledge of available resources and how to access them)
- Avoidance of health care systems and reluctance to seek care (Marshallese only)

**Barriers to social distancing**

- Living in high-occupancy households
- Working jobs where they cannot isolate

• Financial constraints, lack resources or social safety nets (e.g., extended family is not nearby, lack of connections to the local community)

**Abbreviation:** COVID-19 = coronavirus disease 2019.

* All themes apply to both communities unless specified otherwise.

^†^ The outbreak study period (March 11–June 13, 2020) preceded the community engagement study period (June 15–July 3, 2020).

Among a total of 3,436 laboratory-confirmed COVID-19 cases in Benton and Washington counties during March 11–June 13, 647 (19%) occurred among Marshallese persons and 1,554 (45%) among Hispanic persons (Table). Incidences among Marshallese (8,390 per 100,000 persons) and Hispanic persons (1,795 per 100,000) were 71 and 15 times higher, respectively, than incidence among non-Hispanic White (White) persons (118 per 100,000). Approximately one half of COVID-19 cases occurred among males (48% in both groups), and the highest percentage of cases occurred among persons aged 25–44 years (Marshallese, 40%; Hispanic, 35%). Poultry processing^†^ was the most frequently reported occupation among Marshallese (28%) and Hispanic (40%) persons with COVID-19. Overall, 181 (5%) COVID-19 patients were hospitalized across all groups. Compared with the rate of hospitalization in White persons (eight per 100,000), rates were higher among Marshallese persons (765 per 100,000) and Hispanic persons (87 per 100,000); mortality was also higher among Marshallese (130 per 100,000) and Hispanic persons (six per 100,000) than among White persons (two per 100,000). A higher proportion of White persons with COVID-19 were aged ≥65 years (17%) compared with Marshallese or Hispanic persons with COVID-19 (5% aged ≥65 years). However, rates were not age-adjusted because of an absence of accurate population estimates by age for these counties. Analyses of addresses identified 79 households with four or more COVID-19 cases, totaling 390 cases, or 11% of all cases; 35% of persons in household clusters identified as Marshallese and 54% as Hispanic. In 30 (38%) of the 79 household clusters, the initial cases occurred in poultry workers; in the remaining 49 clusters, 18 (37%) included at least one poultry worker with COVID-19.

**TABLE:** Characteristics of persons with laboratory-confirmed COVID-19, by race/ethnicity — Benton and Washington Counties, Arkansas, March 11–June 13, 2020*

Characteristic	No. (%), [rate]^†^				Total
	Marshallese	Hispanic/Latino	White, non-Hispanic	Other, non-Hispanic	
**Population**	7,712**^§^** (2)	86,581 (17)	365,839 (72)	49,437 (10)	**509,569**
**Laboratory-confirmed cases**	647 (19) [8,390]	1,554 (45) [1,795]	432 (13) [118]	803 (23) [1,620]	**3,436 [670]**
**Sex^¶^**					
Female	331 (52)	811 (52)	214 (50)	371 (46)	**1,727 (51)**
Male	310 (48)	738 (48)	217 (50)	427 (54)	**1,692 (49)**
**Age group (yrs)**					
<18	165 (26)	275 (18)	34 (8)	174 (22)	**648 (19)**
18–24	74 (11)	194 (12)	51 (12)	103 (13)	**422 (12)**
25–44	260 (40)	545 (35)	159 (37)	307 (38)	**1,271 (37)**
45–64	118 (18)	464 (30)	115 (27)	179 (22)	**876 (25)**
≥65	30 (5)	76 (5)	73 (17)	40 (5)	**219 (6)**
Employment**					
Poultry work	152 (28)	574 (40)	57 (14)	137 (19)	**920 (30)**
Nonpoultry work^††^	111 (20)	570 (40)	194 (47)	72 (10)	**947 (31)**
Unemployed or retired	76 (14)	105 (7)	36 (9)	13 (2)	**230 (7)**
Unknown	211 (38)	183 (13)	124 (30)	483 (69)	**1,001 (32)**
**Clinical course/outcome**					
Hospitalized	59 (9) [765]	75 (5) [87]	30 (7) [8]	17 (2) [34]	**181 [36]**
Died	10 (2) [130]	5 (0) [6]	8 (2) [2]	3 (0) [6]	**26 [5]**

**Abbreviation:** COVID-19 = coronavirus disease 2019.

* The outbreak study period (March 11–June 13, 2020) preceded the community engagement study period (June 15–July 3, 2020).

^†^ Cases per 100,000 population; rates reported for laboratory confirmed cases, hospitalizations, and deaths.

^§^ 2010 U.S. Census population estimate; this number is assumed to be an underestimate based on reports of school enrollment.

^¶^ Totals for sex do not sum to the total number of cases because of missing data.

** Totals for employment do not sum to the total number of cases because person aged ≤18 years were excluded.

^††^ Nonpoultry work includes all other types of employment (e.g., food service, customer service, health care, construction, self-employed, teaching, and other factory or office work).

Focus group discussions and key-informant interviews revealed that although Marshallese and Hispanic persons were concerned about COVID-19, prevention and mitigation measures were not consistently implemented (Box). High-occupancy households were common in both communities, making quarantine and isolation difficult. Participants reported that staying home from work and seeking medical care were not economically viable. Both groups reported low utilization of medical care. Marshallese persons reported a strong distrust of and anxiety around Western medicine, especially hospitals. Hospital isolation policies and the limited availability of bilingual staff members increased anxiety, confusion, and mistrust.

Participants in both communities reported little awareness of public health messaging and low knowledge regarding SARS-CoV-2 transmission and disease characteristics. Participants also reported being unaware of or unsure about how to access support services available in the local community, leading to confusion around prevention, testing, and services. Participants reported that they typically received information from social networks and on social media. Changing COVID-19 guidance, especially related to reopening, decreased the sense of urgency and increased confusion around the need to continue prevention and mitigation practices. Business owners reported concerns about difficulty enforcing compliance with new guidance. Participants expressed confusion about the meaning and necessity of isolation and quarantine, the difference between the two, and what they needed to do to return to work.

## Discussion

Marshallese and Hispanic communities in two Arkansas counties experienced disproportionate COVID-19–associated morbidity and mortality: COVID-19 incidence, hospitalization rate, and mortality among Marshallese persons were 71 times, 96 times, and 65 times higher, respectively, than rates among White persons. Similarly, COVID-19 incidence, hospitalization rate, and mortality among Hispanic persons were 15 times, 11 times, and three times higher, respectively, than rates among White persons. Disparities in COVID-19 outcomes are likely influenced by long-standing systemic inequities in social determinants of health that have left racial and ethnic minority populations with high rates of underlying conditions (*3*,*4*) and increased risk for COVID-19–associated illness and death (*5*,*6*). Racial and ethnic minority groups are more likely to work where physical distancing is not possible (*5*,*7*) and where COVID-19 incidence is high (*5*) such as within the poultry processing industry, which relies disproportionately on employees from racial and ethnic minority groups (*8*). In addition, high household occupancy is associated with both low income and COVID-19–associated deaths (*5*).

In the United States, low English fluency has been associated with high COVID-19 incidence (*5*,*6*,*9*). Marshallese and Hispanic persons reported a lack of native language information. In addition, Marshallese and Hispanic participants reported limited use of health care systems. Lack of native language messaging from trusted sources (peers, social media, and community and faith-based organizations) in their native languages, low familiarity with health care systems, and an urgent and evolving health crisis combined to create overall confusion regarding prevention, testing, treatment, and availability of support services. The Marshallese community also indicated high levels of preexisting medical mistrust. Current restrictions on hospital visitors, few Marshallese-speaking medical staff members, and an inconsistently available COVID-19 interpretation call-line compounded mistrust, resulting in delayed medical treatment for COVID-19.

To slow community transmission of SARS-CoV-2 in Marshallese and Hispanic communities a number of public health actions, based on focus group input, might increase community buy-in, utilization of health care services, and organizing efforts to slow the transmission of SARS-CoV-2, and decrease duplication of effort. Enhancing coordination of culturally and linguistically tailored outreach, health education, and support services to communities by public health, health care, and community stakeholders might improve the quality and timeliness of information and increase the number of trusted sources who share reliable public health information, leading to increased awareness of risks and adoption of recommended prevention behaviors. Accessible public health communication that does not rely on literacy (in English or native languages), with an emphasis on social media, testimonials, and short videos might increase effective use of information. Beneficial public health topics include factors that can increase or decrease COVID-19 risk and when emergency care should be sought. Also, community partners might be more aware of the social and cultural needs and concerns of the communities and can more closely monitor use of COVID-19 mitigation behaviors, health care, and support services for possible gaps. In addition, policies that allow for workers to miss work for testing, isolation, and quarantine are recommended.

The findings in this report are subject to at least five limitations. First, information regarding underlying medical conditions was incomplete; therefore, epidemiologic analysis in the context of general health status was not possible. Second, the age distribution for Marshallese and Hispanic persons with COVID-19 was younger than that for White persons with COVID-19; controlling for age would likely widen the disparities related to the adverse outcomes of hospitalization and death. Third, self-reported occupation might have led to misclassification of employment. Fourth, in clusters, the initial case was inferred from symptom onset date, specimen collection date, or case report date; therefore, true initial cases might be incorrectly identified. Finally, the Marshallese and Hispanic persons who participated in the focus groups and key-informant interviews might not be representative of their communities.

Communities that suffer disproportionately from COVID-19, especially those affected by long-standing inequities in social determinants of health, need culturally and linguistically tailored public health education, community-based prevention activities, case management, care navigation, and service linkage. Such assistance, paired with a strong coordination of stakeholders, should encourage community acceptance and adoption of prevention and mitigation methods and include opportunities for community feedback to ensure that messaging and services are reaching target populations.

**Summary**

**What is already known about this topic?**

Inequities in social determinants of health have put racial and ethnic minority groups at increased risk for COVID-19 and associated mortality.

**What is added by this report?**

Marshallese and Hispanic persons represented approximately 19% of the population but accounted for 64% of COVID-19 cases and 57% of associated deaths in two Arkansas counties. Contributing factors include lack of relevant health communications, limited coordination between stakeholders, mistrust of the medical system, financial need to work, and household density.

**What are the implications for public health practice?**

Reducing COVID-19 disparities requires strengthening the coordination of public health, health care, and community stakeholders to provide tailored health education, community-based prevention activities, case management, care navigation, and service linkage.

## Footnotes

* 45 C.F.R. part 46, 21 C.F.R. part 56; 42 U.S.C. Sect. 241(d); 5 U.S.C. Sect. 552a; 44 U.S.C. Sect. 3501 et seq.

† Nonpoultry work includes all other types of employment, e.g., food service, customer service, health care, construction, self-employed, teaching, and other factory or office work.
